# Epidemiology of asymptomatic peptic ulcer disease diagnosed during screening endoscopy in patients with cirrhosis

**DOI:** 10.1177/03000605241305258

**Published:** 2024-12-18

**Authors:** Girma Deshimo Lema, Enguday Demeke Gebeyaw

**Affiliations:** 1 School of Medicine, Asrat Woldeyes Health Science Campus, Debre Berhan University, Debre Berhan, Ethiopia; 2 School of Public Health, 227183 Asrat Woldeyes Health Science Campus, Debre Berhan University, Debre Berhan, Ethiopia

**Keywords:** Peptic ulcer, chronic liver disease, liver cirrhosis, asymptomatic, endoscopy, prevalence

## Abstract

**Objective:**

Asymptomatic peptic ulcer disease (PUD) is frequently overlooked in patients with cirrhosis, who are at increased risk for gastrointestinal complications that can lead to increased morbidity and mortality. We aimed to determine the prevalence and associated factors of asymptomatic PUD identified during screening endoscopy in patients with cirrhosis.

**Methods:**

We conducted a retrospective cross-sectional study among patients with cirrhosis at St. Paul’s Hospital Millennium Medical College in Ethiopia. Data were collected using a structured questionnaire from patients’ medical charts. A logistic regression model was used to identify predictors of peptic ulcer.

**Results:**

This study included 296 patients, revealing that 19.6% had PUD (95% confidence interval: 13.5–26.4). Significant associations were found between peptic ulcer and *Helicobacter pylori* infection, moderate and heavy alcohol consumption, and Child–Pugh class C cirrhosis.

**Conclusion:**

We found that patients with liver cirrhosis are more likely to have asymptomatic peptic ulcers. Asymptomatic PUD was linked to *H. pylori* infection, greater alcohol consumption, and Child–Pugh class C liver disease, highlighting the need for targeted prevention and management strategies to reduce morbidity in patients with cirrhosis, such as eradication therapy for *H. pylori* and counseling on alcohol use.

## Introduction

Cirrhosis is a major health problem worldwide and is associated with various complications. Peptic ulcer disease (PUD) is a common concern in patients with cirrhosis.^1^ Studies indicate that patients with cirrhosis have a higher risk of developing PUD than those without cirrhosis. The asymptomatic nature of PUD results in complications leading to morbidity and mortality in patients with cirrhosis. People with bleeding peptic ulcer have a five times greater risk of complications or death than the general population.^2–4^ Peptic ulcer bleeding accounts for 30% of gastrointestinal (GI) bleeding in patients with cirrhosis.^5^

The pathogenesis of peptic ulcer in patients with cirrhosis may be explained by the effect of portal hypertension that causes splanchnic congestion and alters normal reparative processes of the gastroduodenal mucosa. In earlier studies, several changes in the gastroduodenal mucosa among patients with cirrhosis could be explained by a reduced potential difference across the gastric mucosa, impairment of bicarbonate secretion, a reduction in gastric mucosal blood flow, and impairment of gastric mucosal oxygenation.^1,6–10^

The prevalence of PUD among patients with cirrhosis varies across studies, with reports ranging from 11.7% to 39%.^3,7,11,12^ Controversy remains related to factors that contribute to peptic ulcer development in patients with cirrhosis. In the general population, *Helicobacter pylori* infection is central to the pathogenesis of PUD. However, the role of *H. pylori* infection in the pathogenesis of PUD among patients with cirrhosis remains to be elucidated.^5^ Many studies have suggested the involvement *H. pylori* infection in the pathogenesis of PUD in this patient population.^13–15^ However, other studies have found no association between PUD and *H. pylori* infection in patients with liver cirrhosis.^16–20^

A study by Luo et al. indicated that age, male sex, diabetes, chronic renal disease, a history of gastro-esophageal variceal bleeding, and use of non-steroidal anti-inflammatory drugs (NSAIDs) were risk factors for peptic ulcer bleeding in patients with cirrhosis.^21^ On the contrary, Kamalaporn et al. found no significant relationship between peptic ulcer bleeding and age, sex, alcohol consumption, smoking, and the use of NSAIDs.^7^ Instead, those authors found that Child–Pugh classes B and C were independently associated with peptic ulcer.^7^ A multivariate analysis by Calvet et al. revealed that male sex was related to PUD.^22^

Few studies have been conducted in Africa to determine the prevalence of asymptomatic peptic ulcer and risk factors in patients with cirrhosis.^23^ Owing to its asymptomatic nature and lack of clear evidence, screening for peptic ulcer is not routinely conducted among patients with cirrhosis. The disease is diagnosed during endoscopy conducted to identify varices and the cause of upper GI bleeding. To the best of our knowledge, there has been no verified research on the prevalence of PUD and its contributing factors among patients with cirrhosis in Ethiopia. Therefore, in this study, we aimed to assess the epidemiology of asymptomatic PUD in patients with cirrhosis undergoing upper GI endoscopy in variceal screening. Additionally, we sought to identify factors contributing to PUD, which may differ from those in other regions owing to variations in behavioral, sociodemographic, genetic, and nutritional factors.

## Methods

### Study design, setting, and participants

We conducted an institutional-based cross-sectional study at the Gastrointestinal and Endoscopy Unit of St. Paul’s Hospital Millennium Medical College (SPHMMC) in Addis Ababa, Ethiopia. This tertiary referral hospital performs approximately 300 endoscopic procedures (including both upper endoscopy and colonoscopy) monthly for various indications. We included all patients aged 18 years and older with cirrhosis who underwent upper GI endoscopy for the purpose of screening varices from 1 July 2020 to 1 July 2022. This study was conducted from 5 July to 10 August 2022. Patients with incomplete records, recent use of proton pump inhibitors, histamine-2 receptor antagonists, antibiotics, or anticoagulants were excluded, as were those with known hepatocellular carcinoma or existing peptic ulcers.

The reporting of this study conforms to the Strengthening the Reporting of Observational Studies in Epidemiology (STROBE) guidelines.^24^

### Sample size calculation and sampling procedure

The sample size was calculated using a formula for estimating a single population proportion, assuming a 24.7% proportion,^4^ with a 95% confidence interval (CI) and a margin of error of 5%. Based on the abovementioned considerations, the initial sample size was set at 285. The total number of patients with cirrhosis who met the inclusion criteria during the study period was 296. As a result, we opted to include all eligible patients using a convenience sampling approach. This decision was made to ensure the reliability of the data collected, leading to an expanded final sample size.

### Data collection tools and procedures

A pretested questionnaire was used to collect data. The data collection format was designed to capture information from various registries. The questionnaire included sections on background information, behavioral factors, liver disease details, laboratory results, upper GI endoscopy, and risk factors for PUD. Initially prepared in English, the questionnaire was then translated into Amharic. An expert fluent in both languages verified the consistency of the translations.

### Measures

#### Outcome and predictors

The determination of PUD was made based on the results of upper GI endoscopy.

Alcohol consumption was assessed and classified according to the American College of Gastroenterology guidelines, as follows: heavy drinkers, ≥14 drinks per week in men and ≥7 drinks per week in women; moderate drinkers, 7 to 14 drinks per week for men and 4 to 7 drinks per week for women; light drinkers, <7 drinks per week for men and <4 drinks per week for women; never drinkers consumed no alcohol at baseline.^25^

The severity of cirrhosis was assessed according to the Child–Pugh classification. This is based on ascites, which is scored as absent (1 point), slight (2 points), and moderate (3 points); bilirubin scored as <2 mg/dL (1 point), 2 to 3 mg/dL (2 points), and >3 mg/dL (3 points); albumin scored as >3.5 g/dL (1 point), 2.8 to 3.5 g/dL (2 points), and <2.8 g/dL (3 points); prothrombin time prolongation scored as <4 s above the control/international normalized ratio (INR) <1.7 (1 point), 4 to 6 s above control/INR 1.7 to 2.3 (2 points), and >6 s above control/INR >2.3 score (3 points); and encephalopathy: none (1 point), grades 1 and 2 (2 points), grades 3 and 4 (3 points).^26^ The Child–Pugh classification is as follows: class A: 5 to 6 points, class B: 7 to 9 points, and class C: 10 to 15 points.

*H. pylori* infection was determined using a stool antigen test and *H. pylori* serology test.

#### Definitions

Liver cirrhosis was diagnosed using a combination of clinical, biochemical, and imaging studies.^15^ The definition of chronic liver disease (CLD) included cirrhosis and other liver diseases without cirrhosis. An unknown cause of cirrhosis was diagnosed when the etiologic workup was negative with the available investigations.^22^

Peptic ulcer was diagnosed based on the endoscopic findings of a well-defined, round or oval-shaped ulcer crater with a fibrin-covered base in the duodenal or gastric wall.^3^ Asymptomatic PUD was defined as the presence of ulcers in the stomach or duodenum without any noticeable symptoms.^4^

Serum albumin levels were categorized as follows: normal level >3.5 g/dL, mildly low level: 2.5 to 3.5 g/dL, and severely low level: <2.5 g/dL.^27^

### Data processing and analysis

Epi-data version 3.1 was used for data entry and exported to IBM SPSS version 25 (IBM Corp., Armonk, NY, USA) for analysis. We conducted the normality test, model fitness test, and multi-collinearity test. Descriptive statistics such as frequency and percentage were computed for categorical variables. Continuous variables are presented as mean ± standard deviation or median ± interquartile range. To determine the association of different independent variables with the outcome variable, bivariate and multivariate logistic regression analysis were carried out. All variables with a p-value <0.25 in the bivariate analysis were included in multivariate logistic regression analysis. A significant association was considered using odds ratio with a 95% CI and a p-value <0.05.

### Ethical approval and consent

This study was conducted in accordance with the Declaration of Helsinki of 1975, as revised in 2013. The study was approved by the Institutional Review Board of Saint Paul's Millennium Medical College, with reference number PM 23/286 (2 April 2022). All patient information has been de-identified to ensure confidentiality and protect the privacy of participants in accordance with ethical standards. Because this study was a retrospective chart review, patients were not required to provide written or verbal informed consent; this type of study involved the use of pre-existing, de-identified data from the medical records.

## Results

### Sociodemographic characteristics

Among the 296 study participants, there were 212 men (71.6%) and 84 women (28.4%). The median participant age was 35 years, with an age range of 18 to 78 years. Notably, 118 participants (39.9%) were aged between 18 and 30 years. In terms of residence, 174 participants (58.8%) were from rural areas, and 122 (41.2%) were from urban areas (Table 1).

**Table 1.** Sociodemographic characteristics of study participants at SPHMMC, Ethiopia (N = 296).

Variable	Category	Frequency (n)	Percentage
Age, years	18–30	118	39.9
	31–40	84	28.4
	41–50	52	17.6
	51–60	22	7.4
	>60	20	6.8
Sex	Male	212	71.6
	Female	84	28.4

SPHMMC, St. Paul’s Hospital Millennium Medical College.

### Clinical and behavioral characteristics of study participants

In this study, the most common etiology of cirrhosis was hepatitis B virus, with 138 (46.6%) patients affected, followed by alcohol consumption among 56 (18.9%) patients. There were 106 (35.8%), 141 (47.6%), and 49 (16.6%) patients with Child–Pugh class A, B, and C, respectively (Table 2).

**Table 2.** Clinical characteristics of study participants at SPHMMC, Ethiopia (N = 296).

Variables	Categories	Frequency (n)	Percentage
Etiology of cirrhosis	HBV	138	46.6
	HCV	36	12.2
	Alcohol liver disease	56	18.9
	Unknown cause	54	18.2
	Other causes**	12	4.1
Child–Pugh classification	A	106	35.8
	B	141	47.6
	C	49	16.6
Ascites	Yes	178	60.1
	No	118	39.9
*Helicobacter pylori* infection	Yes	175	59.1
	No	121	40.9
NSAID use	Yes	41	13.9
	No	255	86.1
Serum albumin level (g/dL)	Normal (>3.5)	106	35.8
	Mildly low (2.5–3.5)	138	46.6
	Severely low (<2.5)	52	17.6
Variable			
Albumin* (g/dL)	2.00		
Platelet count* (×10^3^/μL)	96		
Total bilirubin* (mg/dL)	1.00		

* Median value. **Other causes include autoimmune hepatitis and metabolic-associated fatty liver disease.

SPHMMC, St. Paul’s Hospital Millennium Medical College; HBV, hepatitis B virus. HCV, hepatitis C virus; NSAID, non-steroidal anti-inflammatory drug.

As shown in Table 3, 127 (42.9%) study participants were never drinkers and 286 (96.6%) were nonsmokers.

**Table 3.** Behavioral characteristics of study participants at SPHMMC, Ethiopia (N = 296).

Variable	Categories	Frequency (n)	Percentage
Alcohol consumption history	Never drinker	127	42.9
	Light drinker	24	8.1
	Moderate drinker	78	26.4
	Heavy drinker	67	22.6
Cigarette smoking history	Yes	10	3.4
	No	286	96.6

SPHMMC, St. Paul’s Hospital Millennium Medical College.

### Prevalence of peptic ulcer disease (PUD)

Of the total study participants, 19.6% (95% CI: 13.5–26.4) were diagnosed with PUD. Among these, 24 participants (8.1%, 5% CI: 4.1–12.8) had duodenal ulcers, 18 (6.1%, 5% CI: 2.7–10.1) had gastric ulcers, and 16 (5.4%, 5% CI: 2–9.5) had both duodenal and gastric ulcers. The ratio of duodenal ulcers to gastric ulcers was 1.3:1. In the duodenal ulcer group, the sex ratio was 1.2:1 whereas in the gastric ulcer group, this was 0.49:1.

### Factors associated with PUD

*H. pylori* infection was found in 59.1% of patients with cirrhosis and 79.3% of patients with PUD. *H. pylori* infection, Child–Pugh class C cirrhosis, a history of alcohol consumption, NSAID use, cigarette smoking, ascites, etiology of cirrhosis, and hypoalbuminemia were significant factors related to the presence of peptic ulcer in bivariate analysis; however, there was no significant association with participants’ age, sex, and occupational status. In multivariate logistic regression analysis, *H. pylori* infection, moderate and heavy alcohol consumption, and Child–Pugh class C were independently associated with the presence of asymptomatic peptic ulcer in patients with cirrhosis (all p* < *0.05) (Table 4).

**Table 4.** Factors associated with asymptomatic peptic ulcer disease among study participants (N = 296).

Variable	Categories	Peptic ulcer (N = 296)		p-value	^a^AOR (95% CI)
		Yes (n)	No (n)		
Etiology of cirrhosis	HBV	26	112		1
	HCV	10	26	0.548	1.34 (0.52–3.47)
	Alcoholic liver disease	8	48	0.097	0.41 (0.14–1.17)
	Unknown cause	10	44	0.628	1.27 (0.48–3.42)
	Other causes*	4	8	0.076	3.67 (0.87–15.42)
Child–Pugh class	A	14	92		1
	B	26	115	0.678	1.21 (0.49–2.97)
	C	18	31	0.041**	3.00 (1.05–8.61)
Alcohol consumption history	Never	14	113		1
	Light	5	19	0.191	2.35 (0.65–8.47)
	Moderate	22	56	0.019**	2.98 (1.19–7.46)
	Heavy	17	50	0.005**	3.79 (1.51–9.53)
Cigarette smoking	Yes	2	8	0.274	2.90 (0.43–19.55)
	No	56	230		1
*H. pylori* infection	Yes	46	129	<0.001**	4.82 (2.19–10.58)
	No	12	109		1
History of ascites	Yes	39	139	0.394	1.42 (0.64–3.14)
	No	19	99		1
NSAID use	Yes	11	30	0.243	1.72 (0.69–4.27)
	No	47	208		1
Serum albumin level	Normal	16	90	0.179	0.49 (0.176–1.38)
	Mild low	26	112	0.240	0.61 (0.27–1.39)
	Severely low	16	36		1

*Other causes including autoimmune hepatitis and metabolic-associated fatty liver disease. **p* < *0.05.

a The model was adjusted for etiology of cirrhosis, Child–Pugh class, alcohol drinking, cigarette smoking, *Helicobacter pylori* infection, history of ascites, NSAID use, and serum albumin level.

AOR, adjusted odds ratio. CI, confidence interval; NSAID, non-steroidal anti-inflammatory drug; HBV, hepatitis B virus. HCV, hepatitis C virus.

## Discussion

According to this study, the prevalence of PUD was 19.6%. The relatively high frequency of asymptomatic PUD in patients with liver cirrhosis is consistent with prior data. The prevalence of PUD among individuals with cirrhosis ranges from 11.7% to 39%, according to the literature.^3,7,11,12^ In a prospective study aiming to determine the frequency of peptic ulcer in different forms of CLD, peptic ulcer was found in 14.7% of patients with cirrhosis;^12^ however, both asymptomatic and symptomatic patients were included and 19% of patients took corticosteroids. Peptic ulcer was identified in 39% of patients in a study by Kamalaporn et al,^7^ which was greater than our data. This variation may be attributed to the higher number of patients with severe liver disease (Child–Pugh class C) in that study compared with our study, as it showed a significant correlation with PUD. In contrast to many other studies, most peptic ulcers detected in our study were found in the duodenum.^17,28^ This could be explained by the fact that drugs are considered the primary risk factor for the development of stomach ulcers. In our study, very few patients had a history of NSAID use. However, another study supports our observation that duodenal ulcers are more common than stomach ulcers.^12^

Regarding the increased prevalence of peptic ulcer in patients with cirrhosis, several changes in the gastroduodenal mucosa may explain this finding, even if the actual cause is not known. These abnormalities can be explained by a reduced potential difference across the gastric mucosa, impaired bicarbonate secretion, reduced gastric mucosal blood flow, and impaired gastric mucosal oxygenation, among other changes.^8,9^

In relation to the severity of liver cirrhosis, Child Pugh class C was associated with peptic ulcer in this study. This is supported by studies reporting that Child–Pugh classes B and C were risk factors for peptic ulcer in patients with cirrhosis.^3,7^ This may be owing to several derangements in the response of the gastroduodenal mucosa to injury and impairment of mucosa protection mechanism in late stages of CLD, which may make patients prone to developing peptic ulcers.^6,7,9^

Because *H. pylori* infection is a known cause of peptic ulcers among individuals without cirrhosis, numerous studies have sought to establish a link between peptic ulcer and *H. pylori* infection in patients with cirrhosis, with varying degrees of success.^14,29,30^ In our study, *H. pylori* infection was a risk factor associated with PUD. This is corroborated by a meta-analysis that found the primary risk factor for peptic ulcers in patients with cirrhosis is *H. pylori* infection.^1^ Calvet et al. also found a positive correlation between *H. pylori* infection and peptic ulcer in this patient group.^22^ However, previously published data^15,16,28^ show no relationship between *H. pylori* infection and PUD in cirrhosis. In our investigation, we found a high prevalence of *H. pylori* infection among patients with cirrhosis (59.1%), in 79.3% of patients with peptic ulcers versus 54.2% in those without peptic ulcers. *H. pylori* infection was found in 20% to 60% of patients with cirrhosis in earlier research.^15,28,29^ Among patients with cirrhosis, factors including decreased bile salt production and impaired gastric mucosal defense owing to portal hypertensive gastropathy may allow *H. pylori* to be more active in the stomach and duodenal bulb, in comparison with individuals who do not have cirrhosis. There may be a correlation between *H. pylori* infection and portal hypertensive gastropathy that contributes to the development of PUD.^10^

Our study revealed a significant association between moderate to heavy alcohol consumption and the presence of asymptomatic peptic ulcers in patients with liver cirrhosis. Other studies did not report a significant association between the prevalence of peptic ulcers and alcohol consumption.^7,22^ Studies have shown that alcohol consumption could be a risk factor or increase the presence of symptoms in patients with peptic ulcer who do not have cirrhosis. The mechanism by which alcohol contributes to the development of peptic ulcers is not fully understood. Nonetheless, alcohol is thought to enhance the production of gastric acid, damaging the protective barrier of the duodenum and stomach, reducing the blood supply to the mucosa, and inducing inflammation, all of which can lead to the formation of ulcers.^31–34^ However, the association between alcohol and peptic ulcers is complex and affected by a number of variables, including the quantity and duration of alcohol use, genetic predisposition, and the existence of additional risk factors like *H. pylori* infection or NSAID use. Further research is needed to better understand this association and underlying mechanisms to determine the optimal strategies for preventing and managing peptic ulcers in asymptomatic patients with cirrhosis who consume alcohol.

As liver cirrhosis advances, portal hypertension may cause splenomegaly, which in turn leads to thrombocytopenia. Although there are few data specifically examining the association between thrombocytopenia and PUD in patients with cirrhosis, it is important to note that thrombocytopenia increases the risk of bleeding. This is particularly relevant in the context of PUD, where bleeding complications can significantly impact patient outcomes.^35,36^ Further research is warranted to elucidate the potential role of thrombocytopenia in the development and management of PUD in patients with cirrhosis.

## Study limitations

There are some limitations in our study. Despite being one of the largest on this topic, this study had a limited sample size, which may affect the generalizability of our findings to larger populations. Additionally, the small sample size constrained our ability to conduct subgroup analyses to distinguish between duodenal and gastric ulcers. Furthermore, because this was a retrospective study, some clinical information was inadequately documented in patients' charts. The retrospective nature of the study also limited our ability to establish causal relationships. Despite these limitations, our findings provide valuable insights regarding PUD in patients with cirrhosis within the Ethiopian context. Future studies with larger sample sizes and prospective designs are necessary to further explore the underlying mechanisms of these factors.

## Conclusions

This study highlights the prevalence of asymptomatic PUD among patients with liver cirrhosis, with nearly one in five participants affected. This finding is clinically important because early identification in these vulnerable patients facilitates effective management and helps prevent potentially life-threatening complications. Therefore, targeted endoscopic screening in patients with cirrhosis may be necessary for diagnosing peptic ulcers and preventing complications. However, further research is needed to assess the cost-effectiveness of this approach. *H. pylori* infection, higher levels of alcohol consumption, and advanced liver disease were all linked to PUD. Targeted interventions, such as eradication therapy for *H. pylori* and counseling on alcohol use, should be prioritized. Further research is warranted to explore the underlying mechanisms of these factors and evaluate the efficacy of preventive strategies in reducing the burden of asymptomatic PUD among patients with cirrhosis.

## Data Availability

The datasets used and/or analyzed in the current work are available upon reasonable request from the corresponding author.
